# The Effect of Extracellular pH Changes on Intracellular pH and Nitric Oxide Concentration in Endothelial and Smooth Muscle Cells from Rat Aorta

**DOI:** 10.1371/journal.pone.0062887

**Published:** 2013-05-17

**Authors:** Verena K. Capellini, Carolina B. A. Restini, Lusiane M. Bendhack, Paulo R. B. Evora, Andréa C. Celotto

**Affiliations:** 1 Laboratory of Endothelial Function, Department of Surgery and Anatomy, School of Medicine, University of São Paulo, Ribeirão Preto, SP, Brazil; 2 Laboratory of Pharmacology, Department of Physics and Chemistry, School of Pharmaceutical Science, University of São Paulo, Ribeirão Preto, SP, Brazil; Thomas Jefferson University, United States of America

## Abstract

**Aims:**

It has been known for more than a century that pH changes can alter vascular tone. However, there is no consensus about the effects of pH changes on vascular response. In this study, we investigated the effects of extracellular pH (pH_o_) changes on intracellular pH (pH_i_) and intracellular nitric oxide concentration ([NO]_i_) in freshly isolated endothelial cells and cross sections from rat aorta.

**Main Methods:**

The HCl was used to reduce the pH_o_ from 7.4 to 7.0 and from 7.4 to 6.5; the NaOH was used to increase the pH_o_ from 7.4 to 8.0 and from 7.4 to 8.5. The fluorescent dyes 5-(and-6)-carboxy SNARF-1, acetoxymethyl ester, acetate (SNARF-1) and diaminofluorescein-FM diacetate (DAF-FM DA) were employed to measure the pH_i_ and [NO]_i_, respectively. The fluorescence intensity was measured in freshly isolated endothelial cells by flow cytometry and in freshly obtained aorta cross sections by confocal microscopy.

**Key Findings:**

The endothelial and vascular smooth muscle pH_i_ was increased at pH_o_ 8.5. The extracellular acidification did not change the endothelial pH_i_, but the smooth muscle pH_i_ was reduced at pH_o_ 7.0. At pH_o_ 8.5 and pH_o_ 6.5, the endothelial [NO]_i_ was increased. Both extracellular alkalinization and acidification increased the vascular smooth muscle [NO]_i_.

**Significance:**

Not all changes in pH_o_ did result in pH_i_ changes, but disruption of acid-base balance in both directions induced NO synthesis in the endothelium and/or vascular smooth muscle.

## Introduction

It has been known for more than a century that pH changes can alter vascular tone and thereby influence the circulation and blood pressure. Gaskell was probably the first to show the importance of pH in modulating the vascular tone. Studying mylohyoid muscle arteries and mesenteric arteries from frog, he demonstrated that acidification increased the vascular diameter while alkalinization decreased this diameter [1]. It has also been known that both extracellular pH (pH_o_) and intracellular pH (pH_i_) can alter vascular tone and that they influence each other [2], [3]. However, the following areas remain unclear: the expected vascular response to pH reduction or augmentation; the mechanisms responsible for pH-induced vasodilation or constriction; whether pH_o_ changes pH_i_; and which of the compartment pH is the major modulator of the vascular tone.

Considering there is no consensus about the effects of pH changes on vascular response we have developed a research strategy to address this issue and have demonstrated that extracellular alkalinization causes endothelium-dependent relaxation through the nitric oxide (NO) pathway in rat aorta [4]. On the other hand, rat aorta response to extracellular acidification is more complex and involves endothelium-independent relaxation through NO and hyperpolarization pathways [5]. In an attempt to better understand these previous findings showing the role of NO in the relaxation induced by changes in pH_o_, the present study was carried out to investigate the effects of pH_o_ changes on pH_i_ and intracellular NO concentration ([NO]_i_) in freshly isolated endothelial cells and freshly obtained cross sections from rat aorta.

## Materials and Methods

### 1. Materials

HEPES, NaOH, nigericin and poly-L-lysine were purchased from Sigma (St. Louis, MO, USA). The probes 5-(and-6)-carboxy SNARF-1, acetoxymethyl ester, acetate (SNARF-1) and diaminofluorescein-FM diacetate (DAF-FM DA) were acquired from Invitrogen (Carlsbad, CA, USA). HCl was purchased from Zilquímica (Ribeirão Preto, SP, Brazil). Thiopental sodium was purchased from Cristália (São Paulo, SP, Brazil). All the other salts were obtained from Vetec Química Fina (Duque de Caxias, RJ, Brazil). All drugs were prepared with distilled water.

### 2. Experimental design

Freshly isolated endothelial cells and freshly obtained cross sections from rat aorta were exposed to pH_o_ changes for analyzing pH_i_ and [NO]_i_ by flow cytometry and confocal microscopy. The experimental protocol was designed to mimic metabolic alkalosis or acidosis, as we have previously done [4], [5]. Then, the extracellular alkalinization was induced by NaOH, whilst the extracellular acidification was induced by HCl. The most popular method for measuring pH_i_ has involved the use of pH-sensitive fluoroprobes [6], and we choose SNARF-1. DAF-FM DA, a selective NO fluorescent probe, was chosen because it exhibits a stable fluorescence intensity in a large range of pH (above pH 5.8) [7].

### 3. Animals

The experimental procedures and animal handling were reviewed and approved by the Institutional Committee for Animal Care and Use of the School of Medicine of Ribeirão Preto, University of São Paulo, and were in accordance with the Directive 2010/63/EU (European Commission). Rats were housed under standard laboratory conditions (12 h light/dark cycle at 21°C), with free access to food and water.

Male Wistar rats (230–280 g) were anesthetized with thiopental sodium (40 mg/kg, intraperitoneal injection), and underwent laparotomy for exsanguination via the abdominal aorta and thoracotomy for thoracic aorta harvesting. The thoracic aorta was carefully dissected free of connective tissue and immediately immersed in Hanks solution (composition [mM]: NaCl 145.0, KCl 5.0, CaCl_2_ 1.6, NaH_2_PO_4_ 0.5, MgCl_2_ 0.5, dextrose 10.0, HEPES 10.0; pH 7.4) to perform cytofluorographic and confocal microscopy analyses.

### 4. Endothelial pH_i_ and [NO]_i_ measurement by flow cytometry

The thoracic aorta, immersed in Hanks solution, was longitudinally opened, and the endothelial cells were isolated by gentle rake friction. The Hanks solution containing the isolated cells was centrifuged at 200 *g* for 2 min, and the cells were resuspended in 1 mL of Hanks buffer. The cells were then loaded with SNARF-1 (10 µM) or DAF-FM DA (5 µM) and maintained in a humidified 37°C incubator gassed with 5% CO_2_ for 30 or 20 min, respectively. The cytofluorographic analysis was performed using a FACScan (Becton-Dickinson, San Jose, CA, USA): the fluorescence was excited with the 488 nm line of an argon ion laser for both dyes, and the emitted fluorescence was measured at 580 nm for SNARF-1 in the acidification experiments, 640 nm for SNARF-1 in the alkalinization experiments, and 515 nm for DAF-FM DA. The fluorescence intensity was evaluated using CellQuest 1.2 software (Becton-Dickinson, Franklin Lakes, NJ, USA). The fluorescence intensity was measured before the unique stimulus and at different time points (t = 1, 3, 9 and 15 min) after the unique stimulus with HCl or NaOH. HCl was used to decrease the pH of Hanks solution containing endothelial cells from 7.4 to 7.0 (final concentration of HCl≈6 mM) and from 7.4 to 6.5 (final concentration of HCl≈10 mM). NaOH was used to increase the pH of Hanks solution containing endothelial cells from 7.4 to 8.0 (final concentration of NaOH≈15 mM) and from 7.4 to 8.5 (final concentration of NaOH≈27 mM); Hanks solution, at pH 7.4, served as control. The fluorescence intensity before the stimulus was designated F_0_, and the fluorescence intensity after the stimulus for each time point was designated F_t_. In this way, the difference in fluorescence intensity (ΔF = F_t_ - F_0_) was obtained for each time point.

Before starting the pH_i_ measurement with SNARF-1, we evaluated the specificity of this dye for acid and alkali stimuli using nigericin and NH_4_Cl at different emitted fluorescence wavelengths (580 and 640 nm), respectively.

### 5. Aorta pH_i_ and [NO]_i_ measurement by confocal microscopy

The fresh aorta cross sections (100 µm thick) were placed on a poly-L-lysine-coated slide. The tissue was loaded with 10 µM SNARF-1 or 5 µM DAF-FM DA and maintained in a humidified 37°C incubator gassed with 5% CO_2_ for 30 or 20 min, respectively. The pH_i_ and [NO]_i_ were assessed using a confocal scanning laser microscope (Leica TCS SP2, Leica Microsystems CMS GmbH, Mannheim, Baden-Württemberg, Germany). The fluorescence was excited with the 488 nm line of an argon ion laser for both dyes, and the emitted fluorescence was measured at 580 nm for SNARF-1 in the acidification experiments, 640 nm for SNARF-1 in the alkalization experiments, and 515 nm for DAF-FM DA. Time-course software was used to capture cross sections images at intervals of 2 s (xyt) in Live Data Mode acquisition at 512×512 pixels at 700 Hz. Aorta cross sections were stimulated at 3^rd^ and 9^th^ min with Hanks acidified or alkalinized solution and cross sections images were captured during 15 min. From 0 to 3^rd^ min the cross sections were immersed in Hanks solution (pH 7.4), from 3^rd^ to 9^th^ min the cross sections were immersed in Hanks acidified (pH 7.0) or alkalinized (pH 8.0) solution, and from 9^th^ to 15^th^ min the cross sections were immersed in Hanks acidified (pH 6.5) or alkalinized (pH 8.5) solution. We used HCl or NaOH to change the pH of the Hanks solution; Hanks solution, at pH 7.4, served as the control. Using the Leica Microsystem LAS AF software (Leica Microsystems CMS GmbH, Mannheim, Baden-Württemberg, Germany), the fluorescence intensity was measured in the endothelial and the smooth muscle layers, separately. The fluorescence intensity before the stimulus (immediately before the 3^rd^ min) was designated F, and the fluorescence intensity at 6^th^ min after the stimulus for each pH value was designated F_pH_. In this way, the difference in fluorescence intensity (ΔF = F_pH_ - F) was obtained for each pH value.

### 6. Statistical analysis

The data are expressed as means ± SEM. The statistical analysis was performed using two-way repeated-measures ANOVA or one-way ANOVA and Bonferroni post-test (Prism 4.0, GraphPad Software, San Diego, CA, USA). *p* values lower than 0.05 were considered statistically significant.

## Results

### 1. Effect of extracellular alkalinization on pH_i_ in isolated endothelial cells

**Figure 1:**
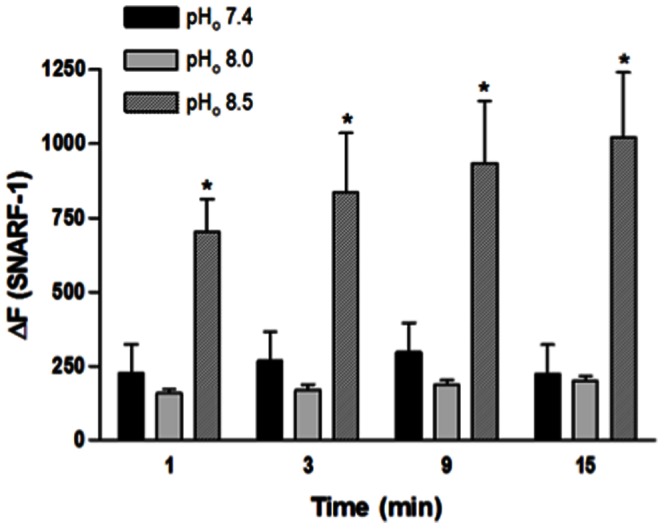
Effect of extracellular alkalinization on pH_i_ in isolated endothelial cells from rat aorta. Cells were loaded with SNARF-1 (10 µM) and analyzed by flow cytometry. NaOH was used to increase pH_o_ from 7.4 to 8.0 and from 7.4 to 8.5; Hanks solution pH 7.4 served as control. Fluorescence intensity was measured before the NaOH stimulus (F_0_) and at different time points (t = 1, 3, 9 and 15 min) after this stimulus (F_t_). Results are reported as ΔF = F_t_ - F_0_. All values are means ± SEM (n = 7). Two-way ANOVA, Bonferroni's post-test, * *p*<0.05 *versus* control.

NaOH-induced extracellular alkalinization increased the SNARF-1 ΔF (pH 8.5 compared with pH 7.4), showing that extreme extracellular alkalinization increased the pH_i_ while less severe pH_o_ augmentation did not change the pH_i_ in freshly isolated endothelial cells (Figure 1).

### 2. Effect of extracellular alkalinization on pH_i_ and [NO]_i_ in aorta cross sections

**Figure 2:**
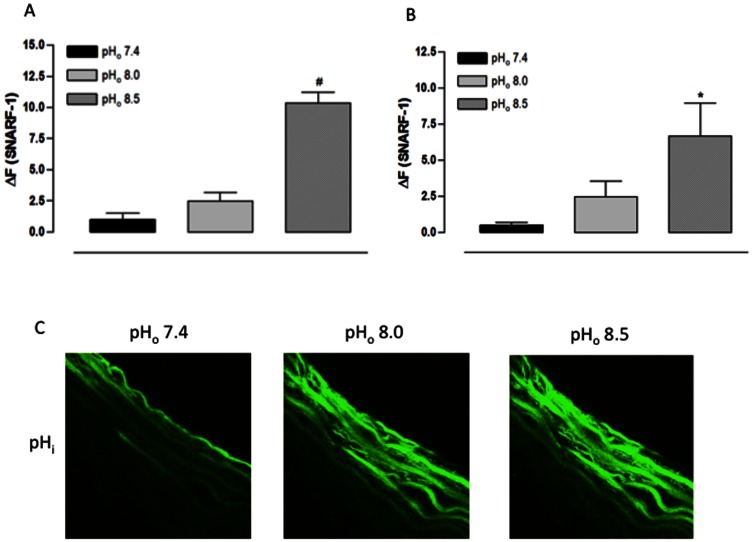
Effect of extracellular alkalinization on pH_i_ of rat aorta cross sections assessed using a confocal scanning laser microscope. A) Fluorescence intensity for endothelial layer. B) Fluorescence intensity for muscular layer. C) Representative confocal photomicrograph of one aorta cross section. Aorta cross sections were loaded with SNARF-1 (10 µM) and analyzed by confocal microscopy. Aorta cross sections were stimulated at 3^rd^ and 9^th^ min with Hanks alkalinized solution pH 8.0 and 8.5, respectively. NaOH was used to change the pH of Hanks solution; Hanks solution pH 7.4 served as control. Fluorescence intensity was measured before the stimulus (F) and at 6^th^ min after the stimulus for each pH_o_ value (F_pH_). Results are reported as ΔF = F_pH_ – F. All values are means ± SEM (n = 7). One-way ANOVA, Bonferroni's post-test, ^#^ *p*<0.001 *versus* control, * *p*<0.05 *versus* control.

Confirming the result above, the pH_o_ 8.5 increased the SNARF-1 ΔF in the endothelial layer of fresh aorta cross sections while the pH_o_ 8.0 did not result in a change (Figures 2 A and 2 C). Furthermore, the result was the same in the muscular layer: the pH_o_ 8.5 increased the SNARF-1 ΔF while the pH_o_ 8.0 did not change it (Figures 2 B and 2 C).

**Figure 3:**
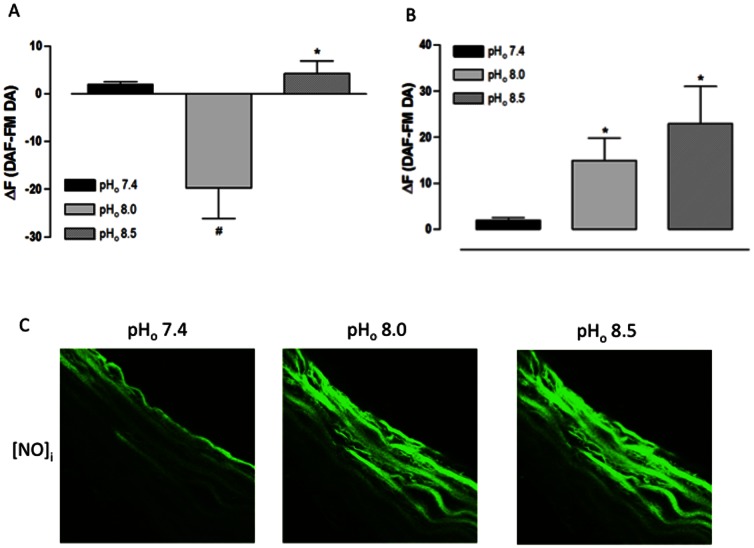
Effect of extracellular alkalinization on [NO]_i_ of rat aorta cross sections assessed using a confocal scanning laser microscope. A) Fluorescence intensity for endothelial layer. B) Fluorescence intensity for muscular layer. C) Representative confocal photomicrograph of one aorta cross section.. Aorta cross sections were loaded with DAF-FM DA (5 µM) and analyzed by confocal microscopy. Aorta cross sections were stimulated at 3^rd^ and 9^th^ min with Hanks alkalinized solution pH 8.0 and 8.5, respectively. NaOH was used to change the pH of Hanks solution; Hanks solution pH 7.4 served as control. Fluorescence intensity was measured before the stimulus (F) and at 6^th^ min after the stimulus for each pH_o_ value (F_pH_). Results are reported as ΔF = F_pH_ – F. All values are means ± SEM (n = 7). One-way ANOVA, Bonferroni's post-test, ^#^ *p*<0.001 *versus* control, * *p*<0.05 *versus* control.

In the endothelial layer of freshly obtained aorta cross sections, the pH_o_ 8.0 reduced while the pH_o_ 8.5 increased the DAF-FM DA ΔF (Figures 3 A and 3 C). However, in the muscular layer, both pH_o_ values increased the DAF-FM DA ΔF (Figures 3 B and 3 C). These data indicate that, at pH_o_ 8.0, a reduction in the endothelial [NO]_i_ was observed simultaneously with an increase in muscular [NO]_i_, and that, at pH_o_ 8.5, the [NO]_i_ increased in both vessel layers.

### 3. Effect of extracellular acidification on pH_i_ in isolated endothelial cells

**Figure 4:**
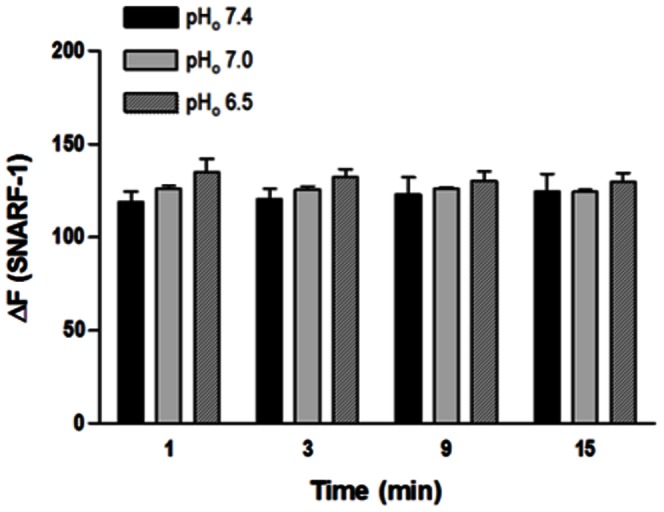
Effect of extracellular acidification on pH_i_ in isolated endothelial cells from rat aorta. Cells were loaded with SNARF-1 (10 µM) and analyzed by flow cytometry. HCl was used to reduce pH_o_ from 7.4 to 7.0 and from 7.4 to 6.5; Hanks solution pH 7.4 served as control. Fluorescence intensity was measured before the HCl stimulus (F_0_) and at different time points (t = 1, 3, 9 and 15 min) after this stimulus (F_t_). Results are reported as ΔF = F_t_ - F_0_. All values are means ± SEM (n = 7). Two-way ANOVA, Bonferroni's post-test.

HCl-induced extracellular acidification had no effect on the SNARF-1 fluorescence, showing that extracellular acidification did not change the pH_i_ in freshly isolated endothelial cells (Figure 4).

### 4. Effect of extracellular acidification on pH_i_ and [NO]_i_ in aorta cross sections

**Figure 5:**
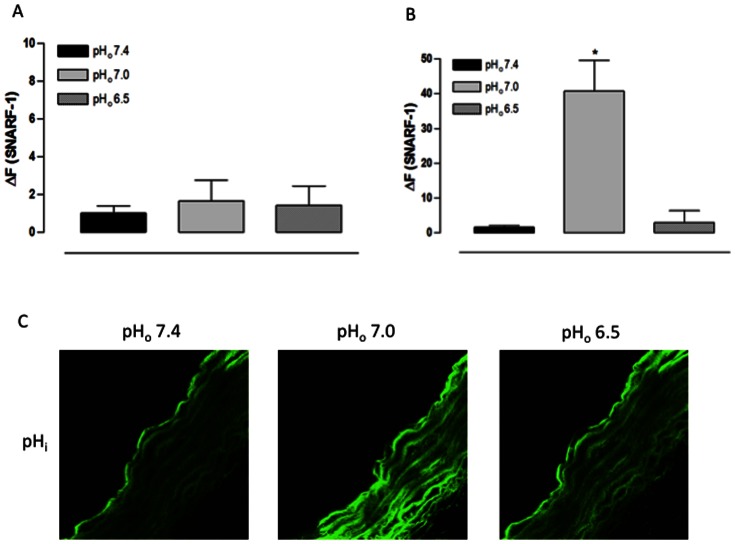
Effect of extracellular acidification on pH_i_ of rat aorta cross sections assessed using a confocal scanning laser microscope. A) Fluorescence intensity for endothelial layer. B) Fluorescence intensity for muscular layer. C) Representative confocal photomicrograph of one aorta cross section. Aorta cross sections were loaded with SNARF-1 (10 µM) and analyzed by confocal microscopy. Aorta cross sections were stimulated at 3^rd^ and 9^th^ min with Hanks acidified solution pH 7.0 and 6.5, respectively. HCl was used to change the pH of Hanks solution; Hanks solution pH 7.4 served as control. Fluorescence intensity was measured before the stimulus (F) and at 6^th^ min after the stimulus for each pH_o_ value (F_pH_). Results are reported as ΔF = F_pH_ – F. All values are means ± SEM (n = 7). One-way ANOVA, Bonferroni's post-test, * *p*<0.01 *versus* control.

Confirming the result above, the SNARF-1 fluorescence in the endothelial layer of freshly obtained aorta cross sections did not change when the pH_o_ was reduced to 7.0 or 6.5 (Figures 5 A and 5 C). However, in the muscular layer, the pH_o_ 7.0 increased the SNARF-1 ΔF while the pH_o_ 6.5 resulted in no change in the SNARF-1 ΔF, showing that the pH_o_ 7.0 acidified the smooth muscle cells pH_i_ which returned to the basal levels with extreme extracellular acidification (Figures 5 B and 5 C).

**Figure 6:**
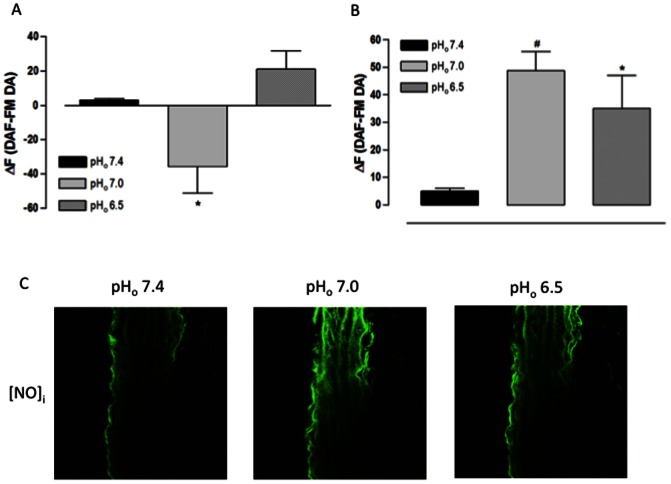
Effect of extracellular acidification on [NO]_i_ of rat aorta cross sections assessed using a confocal scanning laser microscope. A) Fluorescence intensity for endothelial layer. B) Fluorescence intensity for muscular layer. C) Representative confocal photomicrograph of one aorta cross section. Aorta cross sections were loaded with DAF-FM DA (5 µM) and analyzed by confocal microscopy. Aorta cross sections were stimulated at 3^rd^ and 9^th^ min with Hanks acidified solution pH 7.0 and 6.5, respectively. HCl was used to change the pH of Hanks solution; Hanks solution pH 7.4 served as control. Fluorescence intensity was measured before the stimulus (F) and at 6^th^ min after the stimulus for each pH_o_ value (F_pH_). Results are reported as ΔF = F_pH_ – F. All values are means ± SEM (n = 7). One-way ANOVA, Bonferroni's post-test, * *p*<0.05 *versus* control, ^#^ *p*<0.001 *versus* control.

In the endothelial layer of fresh aorta cross sections, the pH_o_ 7.0 reduced the DAF-FM DA ΔF while the pH_o_ 6.5 increased it without statistical significance (Figures 6 A and 6 C). However, in the muscular layer, the DAF-FM DA ΔF was increased with both pH_o_ values (Figures 6 B and 6 C). These data demonstrate that, at pH_o_ 7.0, the [NO]_i_ is reduced in the endothelial layer at the same time in which it is increased in the muscular layer. Moreover, at pH_o_ 6.5, the [NO]_i_ is increased in both vessel layers.

## Discussion

The main findings of the present study are: a) extracellular alkalinization increased the pH_i_ in the endothelium and vascular smooth muscle; b) extracellular acidification did not change the endothelial pH_i_ but reduced the smooth muscle pH_i_; c) severe extracellular alkalinization (pH_o_ 8.5) and acidification (pH_o_ 6.5) increased the endothelial [NO]_i_; and d) both extracellular alkalinization and acidification increased the vascular smooth muscle [NO]_i_.

In the present investigation, we selected aorta because it is the most important conductance artery. In addition, we wanted to clarify previous results obtained with this artery. Another methodological concern that deserves consideration is the use of freshly isolated endothelial cells and fresh aorta cross sections. Studies using freshly isolated cells should be distinguished from those using cultured cells or cell lines, as these cells may no longer express an *in vivo* phenotype [2], [8], [9]. Another important methodological issue is the pH range. We decided to study the pH_o_ from 6.5 to 8.5 because the literature shows that, in this pH_o_ range, the cells are viable [8], [9], [10] and we confirmed this literature data in our previous studies [4], [5].

It is known that both pH_o_ and pH_i_ can alter vascular tone and that they can influence each other. The main factor responsible for this mutual interaction between pH_o_ and pH_i_ is the high H^+^ permeability of arterial vascular smooth muscle. An alternative route would be the flux of weak acids across the membrane. This acid flux can occur either by passive diffusion of the protonated form or via a transporter mechanism. Thus, it is believed that changes in the pH_o_ can induce changes in the pH_i_ in the same direction [2]. However, here we have demonstrated that not all changes in the pH_o_ induce changes in the pH_i_, especially in the endothelium. The extracellular acidification did not produce any change in the endothelial pH_i_ of isolated cells or cells covering the aorta internal surface. Concerning extracellular alkalinization, a great change in the pH_o_ was necessary to induce an increase in the pH_i_ in both isolated endothelial cells and endothelium-intact rings. This suggests that the endothelium, the largest functional organ responsible for regulating vascular tone, coagulation, inflammation and permeability [11], probably has efficient mechanisms of controlling the pH_i_ to avoid changes in enzymatic function and signal transduction, which could impair the important endothelial control of body homeostasis or result in cell death. Moreover, these control mechanisms seem to be more efficient for acidification than for alkalinization. In contrast to the endothelial findings, the extracellular acidification reduced the vascular smooth muscle pH_i_. Actually, at pH_o_ 7.0, the pH_i_ was decreased, corroborating previous data in rat mesenteric arteries [12], [13], cerebral arteries [14] and portal vein [15], but the pH_i_ returned to basal levels when the pH_o_ was reduced to 6.5, probably due to slow recovery to the initial pH_i_ as acid equivalents are removed from the fiber by the pH_i_ regulating mechanism [16]. The vascular smooth muscle exhibited the same behavior as the endothelium when the pH_o_ was increased. Oly extreme extracellular alkalinization increased the pH_i_, which is different from the findings of earlier studies showing that the pH_o_ 7.9 was sufficient to increase the smooth muscle pH_i_ [12], [13], [15]. These variations between endothelial and vascular smooth muscle cells and between distinct vessels can be due to different proton permeabilities or different mechanisms of controlling the pH_i_ [2].

Although the mechanisms by which the pH influences vascular tone remain inconclusive, some evidences suggest the involvement of NO [17], [18], [19], [20], prostacyclin [20], [21], potassium channels [17], [22], [23] and calcium flux [14], [22], [23]. We have previously demonstrated that both extracellular alkalinization and acidification induce vasodilation, and although the mechanisms responsible for this relaxation are not the same in each acid-base disorder, the NO is a common pathway [4], [5]. Here, we have confirmed that changes in acid-base balance did increase the [NO]_i_ in freshly obtained aorta cross sections. However, this [NO]_i_ increase was not necessarily dependent on pH_i_ changes, it could have been due to pH_o_ changes. This observation came from our results showing that, in some cases, even without changing the endothelial or muscular pH_i_, the [NO]_i_ was altered. Moreover, the pH_o_ changes can alter cellular permeability and then activate different signaling pathways [24], [25].

In the present study, we showed that mild extracellular alkalinization (pH_o_ 8.0) and acidification (pH_o_ 7.0) reduced the [NO]_i_ in the endothelial layer and increased the [NO]_i_ in the smooth muscle layer, while severe extracellular alkalinization (pH_o_ 8.5) and acidification (pH_o_ 6.5) increased the [NO]_i_ in the entire vascular wall. The reduction in endothelial [NO]_i_ can be explained by the fast diffusion of this gas to the muscular layer. This event was clearly observed during the confocal experiments, when it was possible to see that the increase in fluorescence intensity in the endothelial layer coincided with the addition of the base or acid, and that, immediately after this addition, the fluorescence intensity in the endothelial layer decreased simultaneously with its increase in the muscular layer. This is confirmed by our previous results showing that the [NO]_i_ in isolated endothelial cells was raised not only at pH_o_ 8.5 and 6.5, but also at pH_o_ 8.0 and 7.0 [4], [5]. Moreover, it was demonstrated that NaOH-induced extracellular alkalinization (pH_o_ 8.0) stimulated the endothelial nitric oxide synthase (eNOS) activity in cultured human pulmonary arterial endothelial cells [8]. Conversely, in cultured human umbilical vein endothelial cells and freshly isolated porcine aortic endothelial cells, the extracellular alkalinization-induced intracellular alkalinization diminished the eNOS activity [26]. Regarding acidosis, it has been shown that the pH reduction predisposes to an increase in the [NO]_i_. In pig cerebellum, the acidification favored the NO synthesis, probably due to an increase in the enzymatic activity [27]. It has also been suggested that, in an acidic milieu, the nitrite can be non-enzymatically reduced to NO [28] and that the NO is more stable in this milieu as it is protected from degradation [19], [29]. However, there are some reports describing that acidification decreases the eNOS activity [8], [26]. Discrepancies between our results (obtained from both extracellular alkalinization and acidification) and this literature data may be attributed to the use of different animal species, different alkali or acid stimulus [2], [24] or different phenotypes (cultured *versus* fresh cells) [2], [8], [9] and the presence or absence of pH_i_ change when the pH_o_ is altered.

An important consideration about [NO]_i_ is that although both extracellular alkalinization and acidification raised the [NO]_i_ in the muscular layer, the NO synthesis was restricted to the endothelium during the augmentation of the pH_o_. On the other hand, during the acidification, the NO was synthesized by the endothelium and smooth muscle. These inferences are based on the fact that the extracellular alkalinization promoted an endothelium-dependent relaxation, which was exclusively mediated by NO [4], while the extracellular acidification-induced relaxation was partially reduced after inhibition of the NO synthesis in endothelium-denuded rings [5]. Another intriguing detail is the endothelial pH_i_ change induced by extracellular alkalinization compared with the lack of change in the endothelial pH_i_ when the pH_o_ was reduced. Based on this observation, would be valid to speculate that this difference is responsible for the exclusive role of NO in the alkalinization-induced relaxation while the vasodilation elicited by extracellular acidification needs factors other than NO?

## Conclusions

In summary, we have demonstrated that not all changes in pH_o_ are reflected in pH_i_ alterations and that both acid-base disorders induce NO synthesis in the endothelium and/or vascular smooth muscle.

## References

[1] Gaskell WH (1880) On the Tonicity of the Heart and Blood Vessels. J Physiology 3: 48–92. 10.1113/jphysiol.1880.sp000083 PMC1484826 16991305

[2] Smith GL, Austin C, Crichton C, Wray S (1998) A review of the actions and control of intracellular pH in vascular smooth muscle. Cardiovasc Res 38: 316–331. 9709392 10.1016/s0008-6363(98)00020-0

[3] Wray S (1988) Smooth muscle intracellular pH: measurement, regulation, and function. Am J Physiol 254: C213–225. 3279796 10.1152/ajpcell.1988.254.2.C213

[4] Celotto AC, Capellini VK, Restini CB, Baldo CF, Bendhack LM, et al (2010) Extracellular alkalinization induces endothelium-derived nitric oxide dependent relaxation in rat thoracic aorta. Nitric Oxide 23: 269–274. 20682356 10.1016/j.niox.2010.07.008

[5] Celotto AC, Restini CB, Capellini VK, Bendhack LM, Evora PR (2011) Acidosis induces relaxation mediated by nitric oxide and potassium channels in rat thoracic aorta. Eur J Pharmacol 656: 88–93. 21300058 10.1016/j.ejphar.2011.01.053

[6] Bond J, Varley J (2005) Use of flow cytometry and SNARF to calibrate and measure intracellular pH in NS0 cells. Cytometry Part A : the journal of the International Society for Analytical Cytology 64: 43–50. 15688357 10.1002/cyto.a.20066

[7] Kojima H, Urano Y, Kikuchi K, Higuchi T, Hirata Y, et al (1999) Fluorescent Indicators for Imaging Nitric Oxide Production. Angewandte Chemie 38: 3209–3212. 10556905 10.1002/(sici)1521-3773(19991102)38:21<3209::aid-anie3209>3.0.co;2-6

[8] Mizuno S, Demura Y, Ameshima S, Okamura S, Miyamori I, et al (2002) Alkalosis stimulates endothelial nitric oxide synthase in cultured human pulmonary arterial endothelial cells. Am J Physiol Lung Cell Mol Physiol 283: L113–119. 12060567 10.1152/ajplung.00436.2001

[9] Nagy S, Harris MB, Ju H, Bhatia J, Venema RC (2006) pH and nitric oxide synthase activity and expression in bovine aortic endothelial cells. Acta Paediatr 95: 814–817. 16801177 10.1080/08035250500462083

[10] Mitchell JA, de Nucci G, Warner TD, Vane JR (1991) Alkaline buffers release EDRF from bovine cultured aortic endothelial cells. Br J Pharmacol 103: 1295–1302. 1884092 10.1111/j.1476-5381.1991.tb09783.x PMC1908361

[11] Crimi E, Taccone FS, Infante T, Scolletta S, Crudele V, et al (2011) Effects of intracellular acidosis on endothelial function: An overview. Journal of critical care 10.1016/j.jcrc.2011.06.001 21798701

[12] Austin C, Dilly K, Eisner D, Wray S (1996) Simultaneous measurement of intracellular pH, calcium, and tension in rat mesenteric vessels: effects of extracellular pH. Biochem Biophys Res Commun 222: 537–540. 8670240 10.1006/bbrc.1996.0779

[13] Austin C, Wray S (1993) Extracellular pH signals affect rat vascular tone by rapid transduction into intracellular pH changes. J Physiol 466: 1–8. 8410686 PMC1175463

[14] Peng HL, Jensen PE, Nilsson H, Aalkjaer C (1998) Effect of acidosis on tension and [Ca2+]i in rat cerebral arteries: is there a role for membrane potential? Am J Physiol 274: H655–662. 9486271 10.1152/ajpheart.1998.274.2.H655

[15] Taggart M, Austin C, Wray S (1994) A comparison of the effects of intracellular and extracellular pH on contraction in isolated rat portal vein. J Physiol 475: 285–292. 8021835 10.1113/jphysiol.1994.sp020069 PMC1160378

[16] Aickin CC (1984) Direct measurement of intracellular pH and buffering power in smooth muscle cells of guinea-pig vas deferens. J Physiol 349: 571–585. 6429320 10.1113/jphysiol.1984.sp015174 PMC1199355

[17] Lindauer U, Vogt J, Schuh-Hofer S, Dreier JP, Dirnagl U (2003) Cerebrovascular vasodilation to extraluminal acidosis occurs via combined activation of ATP-sensitive and Ca2+-activated potassium channels. J Cereb Blood Flow Metab 23: 1227–1238. 14526233 10.1097/01.WCB.0000088764.02615.B7

[18] Gurevicius J, Salem MR, Metwally AA, Silver JM, Crystal GJ (1995) Contribution of nitric oxide to coronary vasodilation during hypercapnic acidosis. Am J Physiol 268: H39–47. 7530920 10.1152/ajpheart.1995.268.1.H39

[19] Hattori K, Tsuchida S, Tsukahara H, Mayumi M, Tanaka T, et al (2002) Augmentation of NO-mediated vasodilation in metabolic acidosis. Life Sci 71: 1439–1447. 12127164 10.1016/s0024-3205(02)01914-8

[20] Zhang Y, Leffler CW (2002) Compensatory role of NO in cerebral circulation of piglets chronically treated with indomethacin. American journal of physiology Regulatory, integrative and comparative physiology 282: R400–410. 10.1152/ajpregu.00256.2001 11792649

[21] Niwa K, Haensel C, Ross ME, Iadecola C (2001) Cyclooxygenase-1 participates in selected vasodilator responses of the cerebral circulation. Circ Res 88: 600–608. 11282894 10.1161/01.res.88.6.600

[22] Dabertrand F, Nelson MT, Brayden JE (2011) Acidosis Dilates Brain Parenchymal Arterioles by Conversion of Calcium Waves to Sparks to Activate BK Channels. Circ Res 10.1161/CIRCRESAHA.111.258145 PMC3505882 22095728

[23] Rohra DK, Sharif HM, Zubairi HS, Sarfraz K, Ghayur MN, et al (2005) Acidosis-induced relaxation of human internal mammary artery is due to activation of ATP-sensitive potassium channels. Eur J Pharmacol 514: 175–181. 15910804 10.1016/j.ejphar.2005.02.041

[24] Celotto AC, Capellini VK, Baldo CF, Dalio MB, Rodrigues AJ, et al (2008) Effects of acid-base imbalance on vascular reactivity. Braz J Med Biol Res 41: 439–445. 18592120 10.1590/s0100-879x2008005000026

[25] Nakamura K, Kamouchi M, Arimura K, Nishimura A, Kuroda J, et al (2012) Extracellular acidification activates cAMP responsive element binding protein via Na+/H+ exchanger isoform 1-mediated Ca(2)(+) oscillation in central nervous system pericytes. Arteriosclerosis, thrombosis, and vascular biology 32: 2670–2677. 10.1161/ATVBAHA.112.254946 22922957

[26] Fleming I, Hecker M, Busse R (1994) Intracellular alkalinization induced by bradykinin sustains activation of the constitutive nitric oxide synthase in endothelial cells. Circ Res 74: 1220–1226. 7514511 10.1161/01.res.74.6.1220

[27] Heinzel B, John M, Klatt P, Bohme E, Mayer B (1992) Ca2+/calmodulin-dependent formation of hydrogen peroxide by brain nitric oxide synthase. Biochem J 281 Pt 3: 627–630. 1371384 10.1042/bj2810627 PMC1130735

[28] Modin A, Bjorne H, Herulf M, Alving K, Weitzberg E, et al (2001) Nitrite-derived nitric oxide: a possible mediator of ‘acidic-metabolic’ vasodilation. Acta Physiol Scand 171: 9–16. 11350258 10.1046/j.1365-201X.2001.00771.x

[29] Ignarro LJ (1989) Endothelium-derived nitric oxide: actions and properties. Faseb J 3: 31–36. 2642868 10.1096/fasebj.3.1.2642868

